# Patient and caregiver experiences with selumetinib for the treatment of pediatric patients with neurofibromatosis type 1 and plexiform neurofibromas

**DOI:** 10.1093/nop/npaf078

**Published:** 2025-08-05

**Authors:** Julia Meade, Michael Blackowicz, Ayo Adeyemi, Randolph de la Rosa Rodriguez, Xiaoqin Yang, Theresa Dettling

**Affiliations:** Pediatric Hematology-Oncology Division, University of Pittsburgh School of Medicine, Pittsburgh, Pennsylvania, USA; Alexion, AstraZeneca Rare Disease, Boston, Massachusetts, USA; Alexion, AstraZeneca Rare Disease, Boston, Massachusetts, USA; Alexion, AstraZeneca Rare Disease, Boston, Massachusetts, USA; Merck & Co., Inc., Rahway, New Jersey, USA; Alexion, AstraZeneca Rare Disease, Boston, Massachusetts, USA

**Keywords:** burden of disease, neurofibromatosis type 1, plexiform neurofibromas, quality of life, selumetinib

## Abstract

**Background:**

Plexiform neurofibromas (PN) affect 20%–50% of patients with neurofibromatosis type 1 (NF1) and can lead to pain, disfigurement, motor dysfunction, compression of vital structures, and risk of malignant degeneration. Selumetinib was the first pharmacotherapy approved for children aged ≥2 years with symptomatic, inoperable PN in the United States and other countries. This qualitative study was conducted to better understand the drivers for initiating selumetinib and the impact of treatment on quality of life (QoL) from the perspective of pediatric patients with NF1–PN and their caregivers.

**Methods:**

The study included pediatric patients in the United States (aged 9–18 years) who had been prescribed selumetinib (≥6 months), and their caregivers. Interviews were conducted, and thematic analyses were performed to identify key concepts. A saturation approach established the point at which no new key concepts were being identified with successive interviews.

**Results:**

Prior to initiating selumetinib, children (*N* = 10) and their caregivers (*N* = 19) reported that PN-related issues, such as pain, impacted the lives of those with NF1–PN. Caregivers played a key role in treatment decisions, and initiation of selumetinib helped meet treatment goals, including PN size reduction, pain improvement, and improved QoL. Patients experienced increased energy, and improvements in pain and all QoL domains post-selumetinib initiation.

**Conclusions:**

Overall, patients and caregivers reported improvements in pain and QoL after selumetinib initiation. The qualitative, real-world nature of this study provides insights into patient and caregiver perspectives, and the impact of selumetinib on the patient journey.

**Key Points**

- Plexiform neurofibromas (PN)-related issues, including pain, can greatly impact the lives of people with neurofibromatosis type 1 (NF1).
- Caregivers play a key role in treatment decisions for children with NF1–PN.
- Selumetinib initiation resulted in improved QoL and pain in children with NF1–PN.

**Importance of the Study**

Neurofibromatosis type 1 (NF1) is a multisystem, genetic disorder. Between 20% and 50% of people with NF1 develop plexiform neurofibroma (PN). PN-related pain can reduce patient quality of life (QoL). Complete surgical removal is often not possible for PN. In April 2020, selumetinib received Food and Drug Administration approval for the treatment of pediatric patients (≥2 years old) with NF1 and symptomatic, inoperable PN. Published qualitative insights into patients’ experiences with selumetinib are limited. To the best of the authors’ knowledge, this is the first real-world qualitative study evaluating selumetinib treatment in the United States from pediatric patient and caregiver perspectives. In this study, patients and caregivers reported improvements in pain and QoL post-selumetinib initiation. Overall, patients and caregivers reported positive experiences with selumetinib that encouraged treatment continuation. The qualitative, real-world nature of this study provides insights into patient and caregiver experiences and the impact of selumetinib on the patient journey.

Neurofibromatosis type 1 (NF1) is a multisystem, autosomal dominant genetic condition resulting from a pathologic variant in the *NF1* gene that causes loss of function of the tumor suppressor protein neurofibromin.^1–3^ NF1 affects approximately 1 in 3000 people globally, with a reported incidence ranging from 1 in 2000 to 1 in 3600 people, depending on study and location.^4–7^ The clinical manifestations of NF1 are varied, and can have a considerable impact on patient quality of life (QoL).^8^ Plexiform neurofibromas (PN) are nerve sheath tumors that affect 20%–50% of patients with NF1, and can cause pain, disfigurement, motor dysfunction, compression of vital structures, and risk of malignant degeneration.^9–14^ Complete surgical resection of PN is rarely possible due to risks such as blood loss, nerve damage, and functional impairment.^15,16^ Therefore, treatment of NF1–PN often focuses on symptom management with careful monitoring/reassessment of tumors.^17^

Selumetinib (ARRY-142886, AZD6244), a mitogen-activated protein kinase kinase 1/2 inhibitor, was approved by the US Food and Drug Administration (FDA) in April 2020 for the treatment of symptomatic inoperable PN in children with NF1 aged ≥2 years.^18^ FDA approval,^19^ and subsequent approval by the regulatory bodies in multiple other countries, was based on the results of the Phase 2 SPRINT trial.^20–22^ Selumetinib demonstrated durable (lasting ≥1 year) PN shrinkage in 56% of pediatric participants and an acceptable benefit–risk profile.^9^ Additionally, clinically meaningful improvements in pain interference and health-related QoL were reported by 38% and 48% of patients, respectively.^9^ At long-term follow-up (up to 5 years of data), selumetinib demonstrated durable PN shrinkage and acceptable benefit–risk profile, as well as sustained improvement in pain.^23^

This qualitative, real-world study aimed to better understand the drivers for initiation of selumetinib treatment, as well as the impact of selumetinib treatment on patient QoL and PN-related symptoms from the perspective of pediatric patients (9–18 years old) with NF1–PN and their caregivers.

## Materials and Methods

Patients and caregivers were recruited using a multifaceted approach, which involved contacting potential participants (via email and/or telephone) who had opted into outreach and advertising on affiliated social media platforms and websites frequented by patients with NF1–PN and their caregivers. A screening questionnaire, which potential participants could complete online or via telephone, assessed eligibility. Potential participants were presented with the study information through a pre-written script. All respondents were re-screened against the eligibility criteria by the recruiting agency.

Inclusion and exclusion criteria for patients and caregivers were considered at the time of screening. Pediatric patients (aged 9–18 years) were eligible for inclusion if they had a diagnosis of NF1, had initiated selumetinib for treatment of symptomatic, inoperable PN, and had been on selumetinib for ≥6 months at screening. Caregivers were eligible for inclusion if they were a parent of, or a primary caregiver (aged ≥18 years) to, a child (aged 2–18 years) who was diagnosed with NF1–PN by a physician, and had been on selumetinib for the treatment of symptomatic, inoperable PN for ≥6 months. All participants were required to understand and speak English. Patients/caregivers were not eligible if they were unwilling or unable to follow study procedures, informed consent was not provided, or the patient was taking selumetinib for a reason other than treatment of symptomatic, inoperable PN.

Patient and caregiver interviews were conducted by a moderator. The moderator was provided with discussion guides for caregivers and for patients. Patients participated in a one-on-one telephone interview of ≤30 minutes long, depending on factors such as their age and ability to express themselves. Caregivers participated in a 45-minute, one-on-one telephone interview. Audio of the interviews was recorded following participant consent. Patients were asked about their lives before and after starting selumetinib, including their experiences with selumetinib, as well as the impact of NF1–PN on their lives. Caregivers were asked for information about their child with NF1–PN and their child’s health condition. These were followed by open-ended questions on their child’s diagnosis and symptoms, their decision to start selumetinib, their treatment goals, their experiences with selumetinib, and the impact of selumetinib treatment on their child’s QoL. Patients and caregivers in the study were not explicitly asked about selumetinib adverse events (AEs) but were made aware that any AEs mentioned in the interview would be officially reported to Alexion for safety purposes. Patients and caregivers who qualified to participate received compensation for their time in the study interviews ($75 and $100, respectively).

To gain a deeper understanding of the patients’ and caregivers’ thoughts and feelings regarding selumetinib treatment, projective techniques, such as associative imagery, were incorporated into the discussion guide. Associative imagery was used to deduce participant descriptions about QoL before and after starting selumetinib. Color photographs and images were shared with the participants over a computer screen, while on the telephone call, and participants selected those that resonated with them based on established research by Gong et al.^24^  Supplementary Table 1 provides descriptions of the 16 images that were shown to participants. This process was used to generate participant responses that would help explain abstract concepts related to their experience with NF1–PN.^24^

The protocol, discussion guide, and all patient communication material were reviewed and approved by the Sterling Institutional Review Board on November 3, 2022. All participants provided informed consent.

A saturation approach was used to establish both a cutoff to the research and an appropriate sample size. Saturation was defined at the point “when no new relevant or important information emerges and collection of additional data will not likely add to the understanding of how patients perceive the concept of interest.”^25^ Saturation grids were developed based on the key discussion topics identified from the thematic analysis, and were used to evaluate the point at which no new key concepts were being identified with each successive interview. Supplementary Tables 2 and 3 depict the saturation grids for patients and caregivers, respectively. Saturation occurs between 9 and 17 interviews.^26,27^

Verbatim transcripts were developed from the audio recordings of interviews; any personally identifiable data were removed, and transcription errors were corrected. Thematic analysis of the data was performed, in which key themes were identified and coded by a qualitative data analyst, supported by qualitative data analysis software (NVivo v12.0) and guided by a flexible coding approach.^28^

Demographic and clinical background data were summarized using descriptive statistics (means, standard deviations [SDs], and frequencies [*n*, %]). Results were descriptive in nature. Graphs and charts were generated using Microsoft Excel.

## Results

### Demographics

The study included 10 children with NF1–PN and their respective 10 caregivers, and another 9 caregivers of children with NF1–PN whose children did not participate directly. The caregiver-reported mean age of patients with NF1–PN was 11 years (*n* = 19, range 3–17 years, SD 4.8 years), and patients had been receiving selumetinib for a mean of 30.6 months (*n* = 19, range 7–96 months, SD 27.6 months). The caregiver mean age was 43 years (range 27–52 years, SD 7.8 years); 14 caregivers were female, and 5 caregivers were male. Further demographics and characteristics of the patients and caregivers are shown in Table 1. A complete list of the quotes captured from patients and caregivers during the interviews can be found in Supplementary Table 4. A full list of caregiver-reported initial signs and symptoms in patients can be found in Supplementary Table 5.

**Table 1.** Caregiver and Patient Characteristics

	Patients, *n* (%), *N* = 19
Patient age subgroup (years)	
2–5	5 (26)
6–12	6 (32)
13–18	8 (42)
Patient duration of selumetinib treatment (months)^a^	
Mean	30.6
Median	18
Range	7–96
Treatment facility^b^^,^^c^	
Academic/university hospital	10 (53)
NF1 treatment center	7 (37)
Community/local hospital	2 (11)
Don’t know	1 (5)
Health insurance^c^	
Private health insurance	13 (68)
Medicaid or other state plan	2 (11)
Insurance purchased through a state exchange	1 (5)
Private health insurance, Medicaid, or other state plan	3 (16)
Other: access to NHS and private medical insurance	1 (5)
Annual household income	
$35 000–$49 999	1 (5)
$50 000–$74 999	3 (16)
$75 000–$99 999	3 (16)
≥$100 000	12 (63)
Caregiver race/ethnicity^d^	
Caucasian/White	10 (53)
Black or African American	6 (32)
Hispanic, Latino, or Spanish origin	4 (21)
Patient school type	
Public school	10 (53)
Private school	7 (37)
Home school	1 (5)
None of the above	1 (5)
Patient grade in school	
Preschool	1 (5)
Kindergarten	2 (11)
1	0
2	0
3	0
4	2 (11)
5	2 (11)
6	2 (11)
7	1 (5)
8	0
9	0
10	3 (16)
11	4 (21)
12	1 (5)
Other	1 (5)

Abbreviations: NF1, neurofibromatosis type 1; NHS, National Health Service.

^a^The individually reported durations of treatment (months) were: 7, 7, 10, 11, 12, 12, 12, 15, 17, 18, 18, 24, 28, 36, 48, 51, 72, 87, 96.

^b^Caregivers may not be able to distinguish between academic/university hospital and NF1 treatment center.

^c^Some caregivers selected more than 1 category for this question.

^d^Race and ethnicity was only reported for caregivers. Race and ethnicity was the same for the child of each caregiver, except for 3 patients and their caregivers. One caregiver reported 2 ethnicities.

### Symptomatic or Clinical Manifestation Burden of NF1 and NF1–PN

Caregivers reported that their children with NF1–PN experienced multiple symptoms or clinical manifestations, which varied in intensity. Thirteen caregivers had extractable answers for what they felt the most bothersome symptom or clinical manifestation of NF1–PN was for their child. PN-related concerns were the most frequently reported response (*n* = 7/13; 53.8%); however, several other responses related to NF1 in general were also stated by caregivers (Table 2). When asked to elaborate on the symptoms that concerned them the most, 1 caregiver noted that, “with the plexiform forming and its location, I was worried about irritation,” while another caregiver noted that they were most concerned about *“*the facial plexiform” and wanted their child to “look normal” and to “halt and not let that progress to more of a deformity.” One caregiver specifically felt concerned about the potential for malignancy and noted that, “these little spots on her skin were growing so fast, I was worried that we were dealing with some kind of cancerous growth.” Three caregivers (23.1%) reported that learning limitations and disabilities were the most bothersome symptom or clinical manifestation. For example, 1 caregiver said, “Just the unknown. It affects people so differently that the unknown was the scary part. We were concerned about her going to school and possibly just falling through the cracks and being in public school and kids possibly making fun of her and things like that,” while another commented on their child’s current situation at school, noting that, “I think he’s doing really well with friends … He had a hard time in public school being accepted because people don’t want to work hard to understand him.” One caregiver commented on their child’s future, saying that, “Life after high school [is most concerning]. Just because of the ADHD, the reading comprehension.”

**Table 2.** Caregiver-Reported Most Bothersome Symptoms and Clinical Manifestations

Symptom or clinical manifestation^a^	*N* = 13 (%)
PN-related concerns	7 (53.8)
Learning limitations and disabilities	3 (23.1)
Disfigured appearance	3 (23.1)
Pain	3 (23.1)
Growth/weight issues	2 (15.4)

Abbreviation: PN, plexiform neurofibroma.

^a^Categories are not mutually exclusive and some caregivers listed more than 1 symptom. Due to the open nature of the discussion guide and interview process, not all caregivers were asked every question.

One patient self-reported being worried about their appearance and their ability to fit in with their peers, and commented on feeling “like no one wanted to be [their] friend back in the day because [they] had a tumor.” Three caregivers (23.1%) reported that a disfigured appearance was the most bothersome symptom or clinical manifestation due to the emotional and social pressures placed on their children regarding their appearance. For example, 1 caregiver reported, “… the one that bothers me the most, is the skin issues. It really bothers me just because it bothers him. It makes him really insecure.”

Pain was the most bothersome symptom or clinical manifestation for 3/13 caregivers (23.1%). They reported that, prior to initiating selumetinib, there were few effective options for pain management, and pain impacted nearly every aspect of the child’s life. Six caregivers described pain as being so intrinsic to the lives of their children that it became normal. For example, 1 caregiver said, “As a parent, it just crushes your heart and your soul to see your kid in pain and know that there’s not much that you can do for it. I can sit and I can rub it and that helps, but it only goes so far … Other pain meds he’s on only go so far.” When asked about pain, 1 patient said, “I got really, really tired because of the pain, or trying to focus harder because of the pain.” Patients and caregivers differed in their reports of the impact of living with NF1–PN. For example, 1 caregiver noted that their child “doesn’t complain” and another stated that their child “doesn’t really mention it like it bothers her or anything.” When discussing the normalization of pain, 1 caregiver stated that, “Pain is always, I think, in the background, like white noise…I think pain is always present for him.”

### Life Before Starting Selumetinib

When asked directly what was bothersome about having NF1–PN, pain was specifically self-reported by 1 patient, who noted that their pain “… would spike sometimes and hurt” and that “just going through that wasn’t the best.” Another noted that, “[the pain is] not very bad, but sometimes it bothers me.” When asked if it was painful, 1 patient described their discomfort by noting that, “[when] standing for a long time [they start] to get tired.” Conversely, another patient expressed indifference to their NF1–PN, noting that, “[they] just don’t really care that [they] have it, but [they] don’t want it.”

When using associative imagery, patients and caregivers were more likely to talk about the impact of NF1–PN with more specificity than they were with direct questioning, and were better able to articulate their experience of pain. For example, 1 caregiver described their child’s life before selumetinib as a burning house, because it conveyed “a lot of destruction and helplessness, and I think that’s exactly where he was,” whereas their child likened their life with NF1–PN to lightning, noting that it was “A lot of pain. Makes me hurt a lot.” Before starting selumetinib, caregivers talked about feeling uncertain, helpless, and out of control because NF1–PN symptoms impacted their child, and symptom management options were limited. For example, 1 caregiver described their child’s life as like a tornado, noting that, “… you see it coming and you can’t stop it. You’re not sure which direction it’s going to go, but you know it’s going to be destructive.” One caregiver identified the picture of a rollercoaster as most similar to their child’s life with NF1–PN due to the “rollercoaster of emotions, like confusion, questioning, even wondering.”

Patients were shown images of lightning, traffic, a rollercoaster, a brick wall, a rainy window, skyscrapers, and a wave. For example, 1 patient selected the lightning image, saying, “It was just a crazy amount of pain.” The images shown to caregivers included a rainy window, lightning, a tornado, a rollercoaster, fireworks, a burning house, a lake and rainbow, red palm trees, traffic, a cactus, blue palm trees, and a brick wall. For example, 1 caregiver selected the rainy window image, saying, *“*It was a couple of years of like, ‘What’s wrong? What’s going on?’ and everything just staying blurry.” Supplementary Table 6 shows descriptions of the images chosen by patients and caregivers before starting selumetinib.

### Patient/Caregiver Journey With Selumetinib

Fourteen caregivers reported the use of at least 1 source of information on selumetinib, and many consulted multiple sources to gather information about the safety and effectiveness of selumetinib. Half of those using sources (*n* = 7/14; 50%) were involved in NF1 online communities hosted through either Facebook or YouTube (Table 3). For example, 1 caregiver said, “Yes, I’m actually on a Facebook group where a lot of people, their kids were participating in either a study or whatever. I was following a couple of different kids and I was seeing the difference and we decided to try [selumetinib] ... Yes, we were following a couple of books on Facebook and yes, I would say I definitely did some research online and stuff.”

**Table 3.** Caregiver-Reported Sources of Information on Selumetinib

Source of information^a^	*N* = 14 (%)
NF1 online communities (eg, Facebook, YouTube)	7 (50.0)
Doctor	6 (42.9)
Clinical studies	5 (35.7)
Search engines (eg, PubMed, Google)	4 (28.6)
Drug manufacturer’s website	1 (7.1)

Abbreviation: NF1, neurofibromatosis type 1.

^a^Categories are not mutually exclusive and some caregivers listed more than 1 source of information. Not all caregivers cited sources of information.

Most of the patients had a more limited role in the initiation of selumetinib. For example, when prompted, 1 patient explained that they were “worried about trying it” but “did not research [selumetinib].” When asked if they remembered anything about the process of starting selumetinib and the decision-making process, 1 patient said that they did “not really” remember, and another said “Honestly, no.” All 19 caregivers described how they decided to initiate treatment with selumetinib (Table 4). For nearly half of the sample (*n* = 9; 47.4%), physicians recommended selumetinib treatment. For example, 1 caregiver said, “[The doctor] called me and she said, ‘I know you were just here; I know they’re recommending surgery. I fully support that if that’s what you want to choose, but I really want you to have the opportunity to learn about selumetinib as a possible option for her.” Approximately half of the caregivers (*n* = 10; 52.6%) had primary roles in advocating for the use of selumetinib; these caregivers were often very active in their child’s treatment as well as in the broader NF1 community. For example, 1 caregiver said, “His oncologist at the time was not well versed in NF or its treatment. He’s primarily a cancer doc. He was honest with us up front. He said, ‘You’re an expert more than I am, and so we’re going to do this together.’ It’s really been a partnership with him. He did advise us that a lot of times these medicines can be difficult as far as dosage and he wanted us to be cautious of our expectations.”

**Table 4.** Caregiver-Reported Reasons for Starting Selumetinib

Reason for starting selumetinib^a^	*N* = 19 (%)
Doctor recommended	14 (73.7)
Shrink PN	6 (31.6)
Exhausted all other options	5 (26.3)
Surgery was too risky	3 (15.8)
Other people experienced success with selumetinib	3 (15.8)
Desire to control pain from PN	3 (15.8)
Clinical reports looked promising	2 (10.5)
Required less monitoring than other treatment options offered	1 (5.3)

Abbreviation: PN, plexiform neurofibroma.

^a^Categories are not mutually exclusive and some caregivers listed more than 1 reason. All caregivers reported a reason.

### Treatment Goals Before Initiating Selumetinib

**Figure 1.**
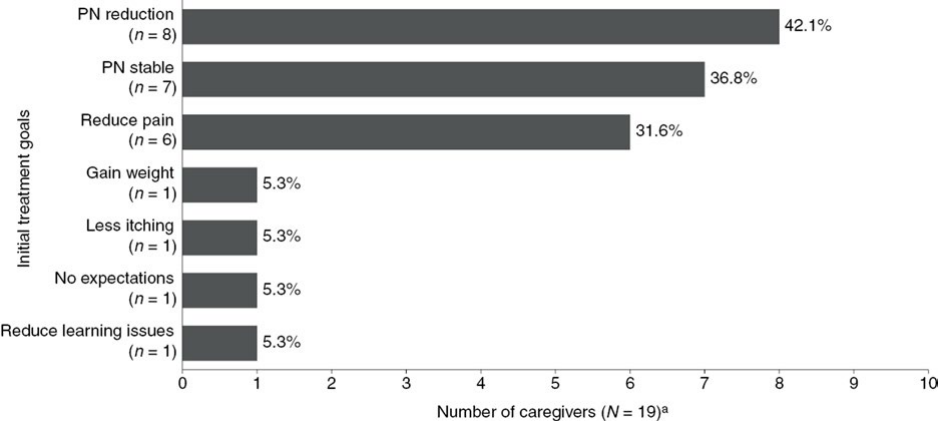
Caregiver-reported initial treatment goals before starting selumetinib. ^a^Categories are not mutually exclusive, and some caregivers listed more than 1 goal. All caregivers responded to this question. PN, plexiform neurofibroma.

All 19 caregivers shared their treatment goals before starting their child on selumetinib. These goals largely centered around reduction in size (*n* = 8; 42.1%) and stabilization (*n* = 7; 36.8%) of their child’s PN (Figure 1). Caregivers also hoped their child would experience less pain from their tumors (*n *= 6; 31.6%). For example, 1 caregiver said, “We wanted no more tumors, but they said that this would be the best option for him to get as close as possible to what his goals were, and which was initially not to be in pain anymore and not to have these tumors everywhere that were growing bigger and bigger,” and another said, “I think it was really about trying to alleviate pain, and stop the growth of the tumor. Just where it is it’s scary in the sense of it causing permanent damage, so we were thinking if we could at least halt any further growth that that would be a step in the right direction.”

### Life After Starting Selumetinib

When asked about how selumetinib improved their pain, 1 patient expressed that they “can sit a lot more longer” and that “basically, when [they] sit it won’t hurt so bad, bugging [them].” Another patient said, “I feel good, I know that I don’t think I’ve had any pain in the area for a very long time.” When asked to elaborate on the main benefits of selumetinib treatment, the same patient noted that, “… just the size and the pain are, I would say, the main ones,” and another patient said, “It’s got a little easier, but it’s stayed the same a bit.”

The impact of selumetinib from a caregiver perspective was varied. For example, 1 caregiver expressed positive feelings toward the effect of selumetinib on their child’s PN, stating that, *“*Monitoring her tumor with the MRIs, it was basically showing that it’s stable, it’s not growing. That’s a win for us. As long as it’s not growing any more, then that’s a success for us.” Another caregiver noted that their child’s PN “immediately shrunk. Actually, within the first month, my husband and I and the oncologist could visibly see that it was smaller ….” In contrast, other caregivers noted that, regarding the impact of selumetinib on PN growth, “It’s been up and down. From there, it’s grown and shrunk each time we visited,” and that they felt, “… a little disappointed because I think we were really, really hopeful that it was going to start shrinking, and that we would see more of the daily benefit of [it]. I think when I feel disappointed, I try to think about if they were growing and there was nothing we could do about it, that would feel worse.”

Overall, caregivers talked about how their child’s life has been more peaceful since starting selumetinib. For example, 1 patient selected the red palm trees image because “It’s way less crazy [the pain] than it was before.” One caregiver selected the blue palm trees image, commenting, *“*Yes. Just more peaceful. He’s more present,” while another selected the rollercoaster image, saying that, “I wouldn’t say his life has changed that much…It’s hard to say. He’s always been a rollercoaster.” A different caregiver selected the cactus image and explained that, “It’s a nice scene, but there are still some prickles (laughs) here and there …We’re in a good place, but it’s never going to be that beautiful paradise.” Supplementary Table 7 shows descriptions of the images chosen by patients and caregivers after the patients started selumetinib treatment.

### Impact on QoL

All caregivers (*n* = 19; 100%) reported some type of improvement in their child while taking selumetinib. Additionally, every caregiver reported some form of physical improvement (*n* = 19; 100%), and about one-third (*n* = 7; 36.8%) reported improvements in the emotional, social, and learning domains. The impact on QoL per theme is summarized in Table 5.

**Table 5.** Caregiver-Reported Impact on Quality of Life by Theme

**PN growth^a^**	***N* = 17 (%)**
PN stable or shrinking	16 (94.1)
Reduced frequency of PN and PN stable or shrinking	1 (5.9)
**Pain^b^**	***N* = 18 (%)**
Pain reduced after taking selumetinib	11 (61.1)
Child did not experience pain	5 (27.8)
Increased pain (unrelated to PN)	2 (11.1)
**Energy^a^**	***N* = 5 (%)**
Exhaustion before taking selumetinib	5 (100.0)
Increased energy after taking selumetinib	5 (100.0)
**Other physical aspects (beyond arresting tumor growth, pain reduction, and increased energy)^c^**	***N* = 9 (%)**
Able to be more physically active	5 (55.6)
Better sleep	3 (33.3)
Better walking	1 (11.1)
Gained weight	1 (11.1)
Improved GI symptoms	1 (11.1)
Improved heart rate	1 (11.1)
Improved strength	1 (11.1)
**Mental and emotional life^c^**	***N* = 10 (%)**
Better self-confidence	7 (70.0)
More calm	4 (40.0)
More capable of dealing with anxieties	2 (20.0)
More expressive	2 (20.0)
**Social and learning domains^c^**	***N* = 6 (%)**
Learning improvement	5 (83.3)
More social with other children	2 (33.3)
Better concentration	1 (16.7)
Better speech	1 (16.7)

Abbreviations: GI, gastrointestinal; PN, plexiform neurofibroma.

^a^These are mutually exclusive categories. Not all caregivers reported PN growth or increased energy.

^b^These are mutually exclusive categories. All caregivers responded to a pain scale.

^c^These are not mutually exclusive categories, and some caregivers listed more than 1 improvement. Not all caregivers reported other physical improvements, improvements to their child’s mental and emotional life, or improvements in social and learning.

When asked if anything had changed in their life since starting selumetinib, 1 patient said, *“*My tumor’s [changed]. It’s gotten smaller... I noticed in the mirror. I was happy because it got smaller.” Seventeen caregivers (89.4%) reported either PN stabilization or reduction since initiating selumetinib treatment and described this change as noticeable. One caregiver commented, “Now that [my child] is taking [selumetinib], you can’t even see [the PN] anymore,” and another noted, “[The biggest benefit has been] the massive reduction in that PN that was visible. It was a reduction in size that was visible to the naked eye and dramatic on MRIs.” On the other hand, 1 caregiver noted that, “Sometimes it grows, and sometimes it shrinks.”

Of the 11 caregivers who reported pain prior to initiating selumetinib, all reported that their child experienced a reduction in pain after taking selumetinib. For example, 1 caregiver stated, “I remember getting up from the table (teary) and crying to my husband in our room because I couldn’t believe he sat at the table and was able to tell us about his school day without having to complain to us that he was in pain.”

Five caregivers (26.3%) reported exhaustion as a symptom of their child’s NF1–PN, and that this improved since initiating selumetinib. For example, 1 caregiver reported, “Yes, his activity level has increased. He’s much more active… He likes to be around kids a lot more… He’s much happier.”

Other improvements to their child’s physical well-being, such as being more physically active, having better sleep and better walking, were reported by 9 caregivers (47.4%), who felt that these improvements all benefitted their child’s QoL. For example, 1 caregiver said that she felt her child was “sleeping a little better, which makes overall mood better,” and 1 patient self-reported, “I can sit there for hours playing my game without having to get up because I’m really uncomfortable.”

Patients described mental and emotional improvements, for example, 1 patient said, “I feel like I can move normally without thinking about I have a tumor or take medicine.” Ten caregivers (52.6%) reported that their child experienced mental and emotional improvements after initiating selumetinib. Of this group of respondents, 7/10 (70%) reported improvement in their child’s self-confidence, manifesting in more social interactions and their child being able to be more independent. For example, 1 caregiver noted, “I think it’s made him have a more positive outlook on life. He’s more confident in being active and associating with others. It makes him less insecure about his appearance and his ability to function. The support is also important as well. All of those have helped him manage his situation.”

Caregivers who reported improvements in the social and learning domains (*n* = 6; 31.6%) saw learning improvements (eg, better reading and less difficulty with schoolwork; *n* = 5; 83.3%) and better concentration (*n* = 1; 16.7%) in their children. One caregiver reported, “Some of [the improvement] is selumetinib… What we thought is, ‘Okay, it takes the pain away. He’s able to concentrate more in school…’” Similar observations were made by patients themselves, with 1 patient self-reporting, “I go to school. I don’t have so much pain anymore. It’s easier to study, and also a lot easier to hang out with my friends.”

### Most Important Benefits of Taking Selumetinib

For the 13 caregivers who were asked about the most important benefits of taking selumetinib, more than half (*n* = 7; 53.8%) reported that the stabilization or reduction in size or frequency of PN was the most important benefit (see Supplementary Figure 1). Several caregivers (*n* = 4; 30.8%) said that a reduction in pain was the benefit they considered the most important. For example, 1 caregiver said, “I have all positive things to say. This was a drug that had enormous benefit… Because it made such a difference in terms of the pain, the growth, all of that… those secondary benefits on the other fibromas were just a bonus.”

### Treatment Goals Since Initiating Selumetinib

To determine whether treatment encouraged optimism about the future, caregivers were asked whether and how their treatment goals had changed since initiation of selumetinib. Of the 11 caregivers who responded, nearly all (*n* = 10; 90.9%) reported that their treatment goals remained unchanged, potentially suggesting belief that these goals could still be achieved. For example, 1 caregiver said, “I’m just hoping that the medication just stays stable because it’s actually helping him stay stable. He’s continuing to improve. As long as there is the same medication, nothing different, same dosages, which is 25 milligrams, or milliliters orally, then I don’t see any changes. I just see him slowly but surely progressing. I see this being a benefit.” One caregiver (9.1%) reported that they were hoping that the progress their child made meant that they could begin to wean them off the medication (see Supplementary Table 4 for the full quote).

## Discussion

This study describes the substantial burden of NF1–PN, with most patients and caregivers reporting multiple symptoms or clinical manifestations of varying intensity. The most frequently reported bothersome symptom was the presence of PN. This finding aligns with previous studies, which have shown that children with NF1–PN have significantly worse health-related QoL than population norms.^29^ Most caregivers in the study presented here reported that their children, with NF1–PN, experienced pain. However, for many, the pain had become such a fundamental part of their lives that it felt “normal” to them and their caregivers. The use of the associative imagery technique^24^ appeared to help patients to articulate their experience of pain.

Half of the caregivers heard about the potential for beneficial impacts with selumetinib through participation in online communities, highlighting the influence of such communities among this small population. However, most caregivers ultimately chose to initiate selumetinib based on physician recommendation, suggesting a shared approach to the decision-making. While PN reduction (42.1%) and stabilization (36.8%) of PN size were the most common treatment goals, some caregivers reported that their primary objective was to address the symptoms associated with NF1–PN, such as pain (31.6%) and learning issues (5.3%).

Based on caregiver responses, selumetinib had a positive impact on patients as they all reported some type of improvement in QoL. Importantly, most caregivers reported PN stabilization or reduction, and a reduction in pain, implying that their key treatment goals were met. More than half of the caregivers reported improvements in the emotional, social, and learning domains. For at least three caregivers, this appeared to result in a greater sense of normalcy for their child. It is possible that some of these benefits may be related to aspects of QoL that were not evaluated in the SPRINT trial, for example, improved self-confidence and social interactions.

There is a growing trend to seek both the pediatric patient and caregiver perspectives on their health and care. These perspectives are important to gain an understanding of subjective experiences, such as pain severity, emotional state, and level of satisfaction.^30,31^ Findings of this study indicated that caregivers observed the overall impact of NF1–PN on patient QoL in greater detail than their child, who were more likely to minimize their NF1–PN symptoms compared with their caregiver. These findings echo those of a previously published study, in which the reporting of symptoms differed between patients and caregivers^32^; results from the current study indicate that patients can underestimate their symptoms. Therefore, the potential to under- or overestimate symptoms should be considered when assessing these results.

One limitation of this study was the small sample size. While small sample sizes are common for rare disease studies, this limited the feasibility of statistical analysis and traditional hypothesis testing. Although the sample size may be considered small by qualitative research standards, the saturation grid approach was sufficient to attain information saturation, based on thresholds in the published literature.^26,27^ Previous research has shown that saturation occurs between 9 and 17 interviews,^26,27^ which is similar to what was observed in this study. Furthermore, the qualitative nature of this study enabled exploration of caregiver and patient experiences, which may not always be elucidated from quantitative research. Additionally, the retrospective nature of the survey may have impacted the ability of participants to accurately recall experiences; the descriptive nature of the results means that the data should be interpreted with caution. The study was also limited by the low number of comments from the patients themselves (and the lower number of patient vs caregiver participants), meaning it may not have been possible to provide a balanced perspective between the patient and the caregiver. Several clinical terms were used in the interviews, which could have been misunderstood by some participants; however, the expected impact of this was reduced by using trained professionals to conduct the interviews and by providing discussion guides to ensure communication on clinical/technical terms was kept as simple as possible. While the use of open-ended discussion during the interview process could have enabled more in-depth insights to be gathered, it may have resulted in varied and inconsistent levels of detail in participants’ responses; thus, leading to some participants not providing extractable answers to some questions. As a result of the open-ended nature of the interviews, not all participants were asked the same questions, potentially introducing bias in data collection. The demographics of the cohort of this study indicated that participants were all from families with an overall household income of at least $100 000 and with a good level of health literacy, demonstrating a potential selection bias in recruitment—the population included in this analysis may, therefore, not be representative of the wider NF1 community, which is considered a study limitation, especially due to the qualitative nature of the results. Furthermore, the nature of the enrollment process could have led to selection bias towards families with a positive treatment experience. Potential participants who were recruited into the study had already consented to receiving advertising and outreach on affiliated social media platforms and website; a more detailed future survey of patients with NF1 and their caregivers from the wider community, which also addresses pain medication use and validated pain scales, would help ensure diversity and inclusion.

This is the first real-world evidence study reporting on selumetinib treatment from the perspectives of both pediatric patients and their caregivers. This research provides evidence that, prior to initiating selumetinib treatment, PN-related issues and PN-associated pain impacted the lives of children with NF1 and their caregivers. Caregivers played a key role in making treatment decisions and typically reported that selumetinib initiation was associated with satisfaction of treatment goals, including tumor reduction, pain improvement, and improved QoL. Patients described increased energy and improvements in emotional, social, and learning domains of QoL after selumetinib initiation. The qualitative, real-world nature of this study enabled explorations of patient and caregiver perspectives and aspects of the impact of selumetinib on the patient journey that may not always be elucidated from quantitative research. Overall, patients and caregivers reported positive experiences with selumetinib that encouraged them to continue with treatment.

## Supplementary Material

npaf078_Supplementary_Tables_and_Figures

## Data Availability

Alexion, AstraZeneca Rare Disease will consider requests for disclosure of clinical study participant-level data, provided that participant privacy is assured through methods like data de-identification, pseudonymization, or anonymization (as required by applicable law), and if such disclosure was included in the relevant study informed consent form or similar documentation. Qualified academic investigators may request participant-level clinical data and supporting documents (statistical analysis plan and protocol) pertaining to Alexion-sponsored studies. Further details regarding data availability and instructions for requesting information are available in the Alexion Clinical Trials Disclosure and Transparency Policy at https://www.alexionclinicaltrialtransparency.com/data-requests/
