# Supplementary material for: Patient and caregiver experiences with selumetinib for the treatment of pediatric patients with neurofibromatosis type 1 and plexiform neurofibromas
Source: Neurooncol Pract. 2025 Aug 5;13(1):86–96. doi: 10.1093/nop/npaf078 (PMC12965642; doi:10.1093/nop/npaf078)
Supplement: npaf078_Supplementary_Tables_and_Figures [file npaf078_supplementary_tables_and_figures.docx]

# **Patient and caregiver experiences with selumetinib for the treatment of pediatric patients with neurofibromatosis type 1 and plexiform neurofibromas**

Julia Meade^1^, Michael Blackowicz^2^, Ayo Adeyemi^2^, Randolph de la Rosa Rodriguez^2^, Xiaoqin Yang^3^, Theresa Dettling^2^

^1^Pediatric Hematology-Oncology Division, University of Pittsburgh School of Medicine, Pittsburgh, PA 15224, USA; ^2^Alexion, AstraZeneca Rare Disease, Boston, MA 02210, USA; ^3^Merck & Co., Inc., Rahway, NJ 07065, USA

**Running Title:** Selumetinib: experiences of people with NF1-PN

**Corresponding Author:** Theresa Dettling, Alexion, AstraZeneca Rare Disease, 121 Seaport Blvd, Boston, MA 02210, USA; Phone number: +1 828.302.8521; Email: Terry.Dettling@alexion.com

# **Supplementary Material**

**Supplementary Table 1.** Descriptions of 16 images shown to participants for the projective associative imagery technique

**Supplementary Table 2.** Saturation Grids for Patients

**Supplementary Table 3.** Saturation Grids for Caregivers

**Supplementary Table 4.** Complete Patient and Caregiver Quotes

**Supplementary Table 5.** Caregiver-reported Initial Signs/Symptoms of NF1, in Addition to PN

**Supplementary Table 6.** Descriptions of the Selection of Images Chosen by (a) Patients and (b) Caregivers Representing Life Before Patients Started Selumetinib

Brief descriptions of the images selected by participants from a choice of 16 images, when asked to describe what daily life was like before starting treatment with selumetinib. Some participants selected more than one image. The table depicts the full list of images selected and the frequency of selection.

**Supplementary Table 7.** Descriptions of the Selection of Images Chosen by (a) Patients and (b) Caregivers Representing Life After Patients Started Selumetinib

Brief descriptions of the images selected by participants from a choice of 16 images, when asked to describe what daily life was like since starting treatment with selumetinib. Some participants selected more than one image. The table depicts the full list of images selected and the frequency of selection.

**Supplementary Figure 1.** Caregiver-reported most important benefits of taking selumetinib.

Caregiver-reported most important benefits of taking selumetinib, out of 13 caregivers who reported a benefit. Categories are not mutually exclusive, and some caregivers listed more than one benefit.

# **Supplementary Tables**

## Supplementary Table 1. Descriptions of 16 images shown to participants for the projective associative imagery technique

| **Picture number** | **Description of picture** |
| --- | --- |
| 1 | Fireworks |
| 2 | Tornado |
| 3 | Rollercoaster |
| 4 | Lightning |
| 5 | Brick wall |
| 6 | Burning house |
| 7 | Lake and rainbow |
| 8 | Red palm trees |
| 9 | Traffic |
| 10 | Cogwheels |
| 11 | Rainy window |
| 12 | Skyscrapers |
| 13 | Wave |
| 14 | Cactus |
| 15 | Forest and stream |
| 16 | Blue palm trees |

## Supplementary Table 2. Saturation Grids for Patients

| **Patient ID** | **PT03** | **PT04** | **PT05** | **PT07** | **PT08** | **PT09** | **PT10** | **PT14** | **PT15** | **PT25** |
| --- | --- | --- | --- | --- | --- | --- | --- | --- | --- | --- |
| **Changes after taking selumetinib** | 1 | 0 | 1 | 0 | 0 | 0 | 1 | 0 | 0 | 0 |
| **Feelings about  NF1-PN** | 2 | 0 | 1 | 0 | 0 | 0 | 3 | 1 | 0 | 0 |
| **How NF1-PN impacts their life** | 0 | 0 | 2 | 1 | 1 | 0 | 1 | 1 | 0 | 0 |
| **Dislikes about having NF1-PN** | 2 | 0 | 1 | 0 | 0 | 0 | 0 | 0 | 0 | 0 |
| **Descriptions of pain** | 1 | 1 | 0 | 0 | 1 | 0 | 0 | 0 | 0 | 0 |
| **Descriptions of taking selumetinib** | 2 | 1 | 1 | 0 | 0 | 0 | 0 | 0 | 0 | 0 |

‘0’ denotes no new concepts were identified in the interview but does not indicate that the respondent did not discuss a concept.

Abbreviations: NF1, neurofibromatosis type 1; PN, plexiform neurofibroma; PT, patient.

## Supplementary Table 3. Saturation Grids for Caregivers

| **Caregiver ID** | **CG01** | **CG02** | **CG03** | **CG04** | **CG05** | **CG07** | **CG08** | **CG09** | **CG10** | **CG14** | **CG15** | **CG16** | **CG17** | **CG18** | **CG19** | **CG20** | **CG22** | **CG23** | **CG24** | **CG25** |
| --- | --- | --- | --- | --- | --- | --- | --- | --- | --- | --- | --- | --- | --- | --- | --- | --- | --- | --- | --- | --- |
| **Experiences prior to initiating selumetinib** | 4 | 1 | 1 | 3 | 0 | 2 | 3 | 2 | 1 | 0 | 0 | 1 | 0 | 0 | 0 | 0 | 0 | 0 | 0 | 0 |
| **Most bothersome symptom** | 2 | 2 | 0 | 0 | 0 | 0 | 1 | 0 | 0 | 0 | 0 | 0 | 0 | 1 | 0 | 0 | 0 | 0 | 0 | 0 |
| **Pain as normal** | 0 | 0 | 1 | 0 | 0 | 0 | 0 | 0 | 0 | 0 | 0 | 0 | 0 | 0 | 0 | 0 | 0 | 0 | 0 | 0 |
| **Role in decision-making** | 1 | 0 | 0 | 1 | 0 | 0 | 0 | 0 | 0 | 0 | 0 | 0 | 0 | 0 | 0 | 0 | 0 | 0 | 0 | 0 |
| **Goals** | 1 | 1 | 1 | 0 | 1 | 1 | 0 | 0 | 1 | 0 | 0 | 1 | 0 | 0 | 0 | 0 | 0 | 0 | 0 | 0 |
| **Sources of information** | 2 | 0 | 0 | 0 | 0 | 2 | 0 | 0 | 0 | 0 | 0 | 0 | 0 | 0 | 0 | 0 | 1 | 0 | 0 | 0 |
| **Reason for starting selumetinib** | 2 | 1 | 0 | 0 | 1 | 0 | 3 | 0 | 1 | 0 | 0 | 0 | 0 | 0 | 0 | 0 | 0 | 0 | 0 | 0 |
| **PN growth** | 1 | 1 | 0 | 0 | 0 | 0 | 0 | 0 | 0 | 0 | 0 | 0 | 0 | 0 | 0 | 0 | 0 | 0 | 0 | 0 |
| **Pain reduction** | 1 | 0 | 0 | 1 | 0 | 0 | 0 | 0 | 0 | 1 | 0 | 0 | 0 | 0 | 0 | 0 | 0 | 0 | 0 | 0 |
| **Increased energy** | 0 | 0 | 1 | 0 | 0 | 0 | 0 | 0 | 0 | 0 | 0 | 0 | 0 | 0 | 0 | 0 | 0 | 0 | 0 | 0 |
| **Other physical improvements** | 0 | 2 | 1 | 0 | 0 | 0 | 0 | 0 | 0 | 0 | 0 | 0 | 0 | 0 | 2 | 2 | 0 | 0 | 0 | 0 |
| **Mental and emotional improvements** | 2 | 0 | 2 | 0 | 0 | 0 | 1 | 0 | 0 | 0 | 0 | 0 | 0 | 0 | 0 | 0 | 0 | 0 | 0 | 0 |
| **Social and learning improvements** | 0 | 2 | 0 | 0 | 0 | 0 | 0 | 0 | 0 | 0 | 0 | 0 | 0 | 0 | 2 | 0 | 0 | 0 | 0 | 0 |
| **Most important benefits of taking selumetinib** | 1 | 0 | 1 | 0 | 1 | 0 | 0 | 0 | 0 | 0 | 0 | 0 | 0 | 1 | 1 | 0 | 0 | 0 | 0 | 0 |
| **Treatment goals since taking selumetinib** | 0 | 0 | 1 | 0 | 0 | 0 | 0 | 0 | 0 | 0 | 0 | 0 | 0 | 0 | 1 | 0 | 0 | 0 | 0 | 0 |

‘0’ denotes no new concepts were identified in the interview but does not indicate that the respondent did not discuss a concept.

Abbreviations: CG, caregiver; PN, plexiform neurofibroma.

## Supplementary Table 4. Complete Patient and Caregiver Quotes

| **Participant type** | **Quote** |  |
| --- | --- | --- |
| **Initial signs and symptoms** |  |  |
| CG | *“She’ll itch the raised ones, especially if they're under her armpit or on the back, they're painful. Then, she is a teeny thing, so she struggles to keep her weight up.”* |  |
| CG | *“What was really a concern for me, the loss of concentration from school, the complaints, and the pain. I tried to get some pain relief at first and tried to encourage him… Yes…the loss of concentration in school was really what got to me, and the pain he complained about.”* |  |
| CG | *“Every time we talked to the doctor about it, they were just like, “No, there’s nothing wrong. There’s nothing wrong. He’s just being sensitive,” because you couldn’t see anything. After a few MRIs, they were just like, “We basically saw all of these plexiforms,” and they’re just like, “This might be what’s causing his pain but there’s nothing we can do about it because of where it’s located.””* |  |
| CG | *“He actually has two plexiforms in his neck. Ironically, one of them has never grown and the other side is just really bad. The one that was removed is the one that has caused the most problems. It was growing in such a way that…it was obstructing his airway. It encapsulates his carotid artery. They believe the plexiform is on the vagus nerve, which is a pretty important nerve in terms of your digestive system.”* |  |
| CG | *“He was just being watched because everything else was pretty normal. At the time, we didn’t see or know about the great big plexiform that ended up forming on his lower back. As that developed, they were watching it more and more and this ended up being a neurofibroma that grew so large it would overhang the waistband of his pants.”* |  |
| CG | *“Based on his clinical symptoms, he had the freckling on his back, on his armpits, and he had the café-au-lait spots. Then what we now saw at that point was a plexiform neurofibroma in his neck. At that point, he was diagnosed clinically, and then I think it was later that year, we actually also did genetic testing to confirm.”* |  |
| CG | *“Of course, I brought up the spots. I was like, “This is a bit weird.” I talked about the pain. I was like, “There’s some pain.” It wasn’t crippling pain. He was in pain, but I could see he was still able to do stuff, move around and do whatever. It was more like discomfort. It wasn’t severe. I was like, “He’s in a bit of pain,” and I was like, “A lot of the pain he’s complaining about is in his back. He complains that his back hurts””* |  |
| **Most bothersome symptom or clinical manifestation – PN** |  |  |
| CG | *“We’ve had good success with the medication in controlling the growth. I would say that is probably the most troubling manifestation for him… I think just the appearance, yes. Just the social concerns.”* |  |
| CG | *“Then post-diagnosis it was probably concerns about discovering where exactly the pain was originating from [the tumors].”* |  |
| CG | *“With the plexiform forming and its location, I was worried about irritation”* | |
| CG | *“These little spots on her skin were growing so fast, I was worried that we were dealing with some kind of cancerous growth”* | |
| **Most bothersome symptom or clinical manifestation – learning limitations and disabilities** |  |  |
| CG | *“Just the unknown. It affects people so differently that the unknown was the scary part. We were concerned about her going to school and possibly just falling through the cracks and being in public school and kids possibly making fun of her and things like that. We just immediately, when she was ready for kindergarten, we put her into a private school, just to try to avoid any conflicts between her and the kids at school.”* |  |
| CG | *“From a social perspective, it’s difficult. He’s at a private school now. I think he’s doing really well with friends. He plays VR, virtual reality. It’s hard when he gets on there. He says a lot of kids call him retarded and say mean things to him because his speech is difficult to understand. He had a hard time in public school being accepted because people don’t want to work hard to understand him.”* |  |
| CG | *“Life after high school [is most concerning]. Just because of the ADHD, the reading comprehension. He doesn’t have a plan yet, and it’s okay but…”* |  |
| **Most bothersome symptom or clinical manifestation – disfigured appearance** |  |  |
| CG | *“This is the one that bothers me the most, is the skin issues. It really bothers me just because it bothers him. It makes him really insecure. I’m not really worried about the heart issues because with the medication, he has high heart rates but now he’s having slower heart rates since he’s been on the medication. That doesn’t bother me as much. He’s at the age now where he’s just really insecure about his appearance.”* |  |
| CG | *“His appearance [the spots], and also his learning and thinking. Right now, he’s in the second grade… His appearance, being in the second grade, there are a lot of kids growing up. They’re just trying to figure out friends and things like that. His appearance really bothers him. He wants to make sure that he looks nice going to school, and school pictures, and everything like that. He has to be on the school bus every morning, every afternoon. His appearance is really a big factor in his life right now.”* |  |
| CG | *“It wasn't so much from a health perspective, but more from… What's the word? Just looking at her. You want her to look normal. (Laughs.) We wanted to halt and not let that progress to more of a deformity.”* |  |
| PT | *“I felt like no one wanted to be my friend back in the day because I had a tumor”*. | |
| **Most bothersome symptom or clinical manifestation – pain** |  |  |
| CG | *“To me, was the ever-growing painful thing on this lower back. For me, until we really got in there and did more scans, it was that nagging uncertainty of, “That’s the one that I can see, what can’t I see?””* |  |
| CG | *“As a parent, it just crushes your heart and your soul to see your kid in pain and know that there’s not much that you can do for it. I can sit and I can rub it and that helps, but it only goes so far. Massages only go so far. Other pain meds he’s on only go so far. To see how that impacted his day-to-day life and he would sit out and just not do stuff, or be as involved…”* |  |
| CG | *“Then she started complaining of the pain and discomfort again. Quite a bit of pain. It was daily or almost daily, especially when she would go to bed. Of course, she stopped being active and then she became very aware of it and said, “We have to do something about this, Mom. It’s interrupting my sleep. I’m so uncomfortable.””* |  |
| PT | *“I got really, really tired because of the pain, or trying to focus harder because of the pain.”* |  |
| PT | *“[The pain] …would spike sometimes and hurt …just going through that wasn’t the best.”* |  |
| PT | *“[The pain is] not very bad, but sometimes it bothers me.”* |  |
| PT | *“[When] standing for a long time [I start] to get tired.”* |  |
| **Most bothersome symptom or clinical manifestation – growth/weight issues** |  |  |
| PT | *“It didn’t really bother me a lot. I remember one time the way that it was phrased, and stuff made me upset, but I was pretty understanding and willing to tell people about it, here.”*  *“Just later when my parents told me about how I may have had a tracheotomy if it hadn’t been treated well, that helped me realize just how blessed I am to have gotten the medical treatment.”* |  |
| CG | *“My biggest concern is the weight, and then the doctor always said [the tumors] are benign, but I’m afraid… What if it turns to not being benign? I know he told me that that doesn't happen. I’m just nervous about the fact that it’s like a tumor.”* |  |
| CG | *“Oh gosh. I don’t know if it’s the pain or looking different. When his legs were such different sizes, he would get stares at the store, or at school. That would just set him apart. He would comment, “I want my knees to be the same size…” As that plexiform was shrinking he was like, “My knees are getting to be the same size!” I was like, “That must be really important to him for him to say that,” and I never knew.”* |  |
| **Living with NF1-PN – PN size** |  |  |
| CG | *“Our understanding from all the doctors we met with which was an ENT, a neurologist, a neurosurgeon, a geneticist, they all said, “We’re looking at a tracheotomy. He’s not going to be able to breathe. It’s going to choke him.” We shielded him from all of that. We did not talk to him about that. That’s why we went and met with a counsellor, a family therapist, like, “Y’all, this is not in a parenting book. What do we do?””* |  |
| **Living with NF1-PN – pain** |  |  |
| PT | *“It was just the pain. It wasn’t making the entire day bad and all. Also, I got really, really tired because of the pain, or trying to focus harder because of the pain.”*  *“I go to school. I don’t have so much pain anymore. It’s easier to study, and also a lot easier to hang out with my friends.”*  *“I think it’s had a lot of improvement. Whenever I play games with my friends, or talk to them, it’s just easier to talk to them, hang out with them. We’ll work on stuff without the pain and getting tired from the pain.”* |  |
| CG | *“He wouldn’t take his power chair to school every day. He had a walker too at that time. He might take the walker instead of the power chair. He would go and you would ask him, “What did you do at recess?” “I did this. I did this.” Before, he might be like, “I just watched my friends play,” or, “We hung back and did something else.” Gosh, within the first 6 weeks I would say that we had pain diminishing. Actually, it was probably within the first 4 weeks because he’s really good…”* |  |
| **Living with NF1-PN – pain and inconvenience** |  |  |
| PT | *“It’s okay sometimes. [But sometimes] it can be difficult because from the pain.”* |  |
| PT | *“I think the thing that bothered me the most was just treatment for it. Missing a lot of school and stuff was rough.”* |  |
| PT | *“It was just the pain. It wasn’t making the entire day bad and all. Also, I got really, really tired because of the pain, or trying to focus harder because of the pain.”* |  |
| PT | *“It feels like I cannot be fully physical. Also, I think that this is making my toe not to be able to point down really compared to my right foot.”* |  |
| PT | *“[Having] NF1 is annoying because I have to take meds a lot… Twenty different pills per day.”* |  |
| **Living with NF1-PN – not noticing/not being bothered** |  |  |
| PT | *“Nothing really bothers me about it.”* |  |
| PT | *“I don't notice [it].”* |  |
| PT | *“Not much. Nothing [bothers me about having NF1] … I just don’t really care that I have it [tumor], but I don’t want it.”* |  |
| **Living with NF1-PN – trying to be normal** |  |  |
| CG | *“For the most part he always tries to be as normal as possible, and those are his words, not mine. He always says, “I just want to be normal. I just want to be normal.” He tries 100 percent of the time to be prepared for the next day. If he knows what’s going on the next day, he will research, he will do whatever he needs to do so that he’s at the same speed as everyone else or close to, so that he doesn’t feel like he’s any different from anybody else.”* |  |
| CG | *“I would say she’s in overall great health. The NF1, I would say it’s affected her in school. She does have some issues with staying focused in class. She struggles with math. Really, she’s generally lived a pretty normal life.”* |  |
| CG | *“Okay… I can remember we got into an after-school program…and one day the director called me and said, “Hey, your kid just told me he has a tumor. We’re one of those places where we want to care on y’all. Like, what? Can you tell me more?” I was like, “What do you mean?” She said, “When he walked in, we said, “Hey, we missed seeing you yesterday.” “Oh, I had an MRI for my tumor,” and he ran into the gym. She’s like, “Wait a minute. Come back.” He was just so matter of fact about it. He didn’t really understand what that meant at that point. He just knew that he had to see a lot of doctors and things. We told him what to tell us. “If it hurts, you need to tell us, buddy.” “Okay,” and that was it.”* |  |
| CG | *“He goes with the flow. He’s very outgoing. He’s very social which is what his doctors have told us is very unusual for patients with neurofibromatosis.”* |  |
| CG | *“[My child] is not a person of very many words. His answer might be, “I don’t know,” because honestly, it’s also like I said, if I say, you need to take this, it’s going to help you, he’s going to say, “Okay” and he’s not going to give it another thought. He’s going to think, “I need to take this because it’s going to help me” and he knows we’re not going to give him anything that he shouldn’t have. I don’t know how he would answer it. He may just be like, “It’s fine.””* |  |
| CG | *“The other piece of that was I wanted my daughter to grow in confidence that this is just part of her and it’s not a negative component of her life. At a very young age, she embraced “This is my reality.” She talks freely about it. It’s just part of her life. Sure, it’s different than her friends, but she just explains it to them, and then they’re very supportive of her and her journey.”* |  |
| **Living with NF1-PN – difficulty expressing feelings and worries** |  |  |
| CG | *“He sees a therapist to talk through things. I don’t believe he’s got anxieties or worries about his NF. I think it’s just about the fact that he has to do more than other kids. He has to work harder than other kids. He has to do more. Summer can’t just be summers without still getting speech and OT and different therapies that he has. He realizes those differences. That frustrates him, I think, but I don’t believe there are any worries about the NF itself.”* |  |
| CG | *“I think my biggest struggle with NF is that we never know what’s next. Some people think, “You got it out of the way early,” and I’m like, “No, another something…” Waiting for the other shoe to drop has definitely made me more anxious as a person.”* |  |
| **Living with NF1-PN – normalizing NF1-PN** |  |  |
| PT | *“Probably just try and ignore it [pain] and have fun playing with friends.”* |  |
| CG | *“Basically, we didn’t understand the pain he was in until he started [selumetinib] because a couple months into taking [selumetinib], he finally said, “I’ve always had this pain and I just thought it was normal but now it’s like a load of elephants are off my back. I don’t feel so tired, I don’t feel all these things. It’s so weird not to feel what I’ve been feeling all this time.” We don’t know how long he’d been in pain because, to him, this was normal.”* |  |
| CG | *“[She does] not really [communicate with me about having NF1-PN]. It’s been a part of her life this whole time. No, she doesn’t really mention it like it bothers her or anything.”* |  |
| CG | *“He doesn’t complain. Even if he did hurt, I don’t know that he would say anything.”* |  |
| CG | *“He was impacted a lot just living day-to-day. Pain is always, I think, in the background, like white noise. You don’t hear it unless somebody turns up the volume, and then white noise becomes annoying. I think pain is always present for him.”* |  |
| CG | *“How do you know what your limitation is if you don’t know what it is without a limitation?”* |  |
| CG | *“She’s on gabapentin [to manage pain]. That helps a little bit, but honestly, once she started the new medication, after a couple weeks she started feeling a little bit better, and she’s able to at least try more. I guess she feels like maybe now she’s being listened to. When she would say, “I hurt,” and I can’t figure out how to help her, at least now she knows, in her mind, we’re trying to help, which we were trying to the whole time.”* |  |
| **Living with NF1-PN – appearance** |  |  |
| PT | *“The tumor itself doesn't affect me a ton other than— I remember when I was young, it used to be super visible… It didn’t really bother me a lot. I remember one time the way that it was phrased, and stuff made me upset, but I was pretty understanding and willing to tell people about it, here.”* |  |
| PT | *“I felt like no one wanted to be my friend back in the day because I had a tumor.”* |  |
| CG | *“That’s been the biggest help. That IP in the school system is literally a piece of paper. You don’t enforce it, and you care less about it. I think it's the therapy that helps the most. I would say that has been the biggest... Because what the NF1 does to girls, a lot, is it affects their self-esteem.”* |  |
| CG | *“He’s been increasingly, as he’s gotten older the last couple of years, I would say he’s been increasingly concerned about if it’s going to cause issues for him socially, if it’s going to affect his acceptance with friends and those kinds of things. He asked his mom just a couple of weeks ago if she thought that it would be likely that he would have a girlfriend later on because of his tumor. He was worried about that. Just those concerns. If the visual appearance of it is going to be a barrier for him.”* |  |
| CG | *“Yes because a year ago he had a school play. It was around Halloween. They were going to do something for Halloween for the kids. He didn’t want to participate because he didn’t feel good about himself. His appearance wasn’t right. He had marks on his body. However, last year in October, he did participate in the Halloween program with the kids. Yes, he felt very good. He felt very confident about himself and did a great job.”*  *“I spoke to a doctor. They said, “A kid can live a normal life.” He’s not so concerned about that. It’s just when he gets older towards his teenage years and girls are coming around and he wants to get into girls, he’s a little worried about that. “The girls don’t look at me. I’ve got spots on my body. Are they going to think I’m sick?” That’s his only concern at this point.”* |  |
| CG | *“Honestly, I think his biggest comments that have gotten to him are, he has about 90 café-au-lait spots, and so when we go to the pool at the Y, the little kids ask him why he’s polka dotted.”* |  |

| **Image selection – life before starting selumetinib** |  |
| --- | --- |
| PT | Rainy window, picture 11: *“Just because it’s raining and gray and maybe sad in a way. It’s not sunny like happy, because of the pain that I had.”* |
| PT | Lightning, picture 4: *“It was just a crazy amount of pain.”* |
| PT | Lightning, picture 4: *“A lot of pain. Makes me hurt a lot.”* |
| PT | Wave, picture 13: *“I thought it was a tsunami, waves building up and getting bigger and my tumor was getting bigger and bigger, so it was going to— It was a threat to me. If it had kept growing, to cut it out, they would have had to give me— I would have had to have a tracheotomy or something and be tube fed because it’s on my carotid artery.”* |
| PT | Brick wall, picture 5: *“Because it looks boring. Because I wasn't really doing much, and now my life is just like normal and back then, it felt like it was boring.”* |
| PT | Skyscrapers, picture 12: “*I think 12 mainly because it reminds of [going to the hospital] because I go there a lot with it.”* |
| CG | Rainy window, picture 11:*“It was a couple of years of like, “What’s wrong? What’s going on?” and everything just staying blurry.”* |
| CG | Rainy window, picture 11: *“It just looks sad… He’s like, “I’m stuck inside. I want to go outside and play. I can’t go outside and play. I’m stuck in here.” Not able to do what he wants, just this overwhelming cloud of pain and itching.”* |
| CG | Rainy window, picture 11: *“I picked that picture because my son, even though it’s only rain, he felt something coming on. He knew something wasn’t right. It’s like an impending storm.”* |
| CG | Rainy window, picture 11: *“Eleven makes me think of that unknown, that uncertainty.”* |
| CG | Lightning, picture 4: *“I attribute the electricity to the nerve pain because I know that those tumors are growing along her nerve sheath. I know that’s what’s causing pain, so I attribute the electricity to the pain impulse.”* |
| CG | Rollercoaster, picture 3: *“I’m guessing a roller coaster of emotions, like confusion, questioning, even wondering. Kids with learning disorders always know that they’re different, so confusion, like, “Why don’t I grasp this?”* |
| CG | Tornado, picture 2: *“…you see it coming and you can’t stop it. You’re not sure which direction it’s going to go, but you know it’s going to be destructive.”* |
| CG | Tornado, picture 2: *“Before starting treatment, I would say number two, the tornado. I think that’s a tornado, it’s a little bit hard to tell. Just because it did feel like this big storm that we were trying to get ahead of, and trying to get control of, so to speak.”* |
| CG | Burning house, picture 6: *“I feel like six shows a lot of destruction and helplessness, and I think that’s exactly where he was.”* |
| **Image selection – life after starting selumetinib** |  |
| PT | Red palm trees, picture 8: *“Some days it’s like eight, really calm.”* |
| PT | Red palm trees, picture 8: *“Because it’s way less crazy [the pain] than it was before.”* |
| PT | Rollercoaster, picture 3: *“Because it's a rollercoaster. Might have been in between [good and bad]. It can be good. It can be bad. I get to go places that some kids wouldn't. It's because I have NF. [The bad is] probably just some of the stuff I'm probably going to have to do for the rest of my life, and it's just a path, walking the way.”* |
| PT | Blue palm trees, picture 16: *“Because it looks relaxing, not a lot of pain… Basically, when I sit it won’t hurt so bad, bugging me.”* |
| PT | Blue palm trees, picture 16: *“Sixteen right now because in the beginning it might be 14 because the first 6 months on the treatment, side effects were really harsh. It was getting better. After 6 months, the clouds parted, and I just stopped having the severe side effects.”* |
| PT | Fireworks, picture 1: *“Because of how good [selumetinib] has been and how it has resolved basically everything. That is just, I feel good, I know that I don’t think I’ve had any pain in the area for a very long time.”* |
| GC | Rollercoaster, picture 3: *“I wouldn’t say his life has changed that much… It’s hard to say. He’s always been a rollercoaster. I’d pick a rollercoaster.”* |
| CG | Rollercoaster, picture 3: *“Celebration that she’s able to get up and move around.”* |
| CG | Fireworks, picture 1: *“Fireworks. Yes. Celebration, like, “Woohoo!””* |
| CG | Blue palm trees, picture 16: *“Yes. Just more peaceful. He’s more present.”* |
| CG | Traffic, picture 9: *“I would say a bit of nine because she’s trying to navigate it now. With treatment, she’s trying to get through it.”* |
| CG | Cactus, picture 14: *“It’s a nice scene, but there are still some prickles (laughs) here and there. You still have, it’s not completely free of pokes and a couple of turns, but it’s peaceful, it’s a better place. We’re in a good place, but it’s never going to be that beautiful paradise.”* |
| CG | Lightning, picture 4: *“I would say since then, probably number four, because it feels like with it starting to grow again, it’s still stormy, but it’s not as intense as the tornado picture.”* |
| **Role in decision-making** |  |
| CG | *“First of all, I’m a believer in letting the experts do their jobs, so if I have this neurologist that is able to diagnose my kid on the first visit, what’s wrong? He was able to diagnose him. I’m like, “This dude clearly knows what he’s doing. If he recommends that this medication can help, then why not go for it?” So, I was like, “Of course.””* |
| CG | *“Sure. His oncologist at the time was not well versed in NF or its treatment. He’s primarily a cancer doc. He was honest with us up front. He said, “You’re an expert more than I am, and so we’re going to do this together.” It’s really been a partnership with him. He did advise us that a lot of times these medicines can be difficult as far as dosage and he wanted us to be cautious of our expectations.”* |
| CG | *“I am heavily involved with the Children’s Tumor Foundation. I am on their volunteer leadership board, and I have been working with, volunteering for, advocating with, since he was diagnosed. This is back, pre-FDA approval for NF1, all of that. I knew it was a possibility in the works, but when we found his tumor, our NF specialist basically said, “We have two options, we can put you on a traditional chemo regimen where you come in once a week and the whole nine yards, or we can try this other treatment.””* |
| CG | *“[The doctor] called me and she said, “I know you were just here; I know they’re recommending surgery. I fully support that if that’s what you want to choose, but I really want you to have the opportunity to learn about selumetinib as a possible option for her.””* |

| **Involvement of child in selumetinib initiation** | | | |  |  |
| --- | --- | --- | --- | --- | --- |
| CG | | | | *“She didn’t really have much [to say]. We just made a family decision…like I said earlier, she just rolls with the punches, you know?”* |  |
| CG | | | | *“Yes, he was in the room. When they talked to me and explained it to me, they also talked to him and explained it to him in words that he would understand. They spent probably more time talking about it and explaining to him all the side effects, all the things that could happen, the positives and the negatives, and they ultimately said that “Even though your mom’s in charge, we want to hear what you feel too before we all agree what’s your best next step.” He just said, “I want to take the medicine because I don’t want to be in pain anymore.””* |  |
| CG | | | | *“He had lots of questions. He is my kid. Trying to explain, what is a MEK inhibitor. From what I could learn and what the doctor said, and asking good questions in front of his doctor because she gives really perfect answers that are digestible to a degree possible for him as a preteen, she’s just spectacular. Being very upfront with him about, “This is what side effects they have recorded. That doesn’t mean you’re going to get them, but we don’t know.” Basically, getting him ready for, “This could not work at all, or this could work,” and trying to explain that. He was very familiar with the concept of clinical trials after his dad’s illness.”* |  |
| CG | | | | *“No. [We did not ask his opinion.]”*  *“No, [he did not have any questions about it.] He just is so, “Mom and dad said to do this, so I’m going to do it.””* |  |
| CG | | | | *“I think he’s thankful for it [the treatment], especially nowadays when he goes off of it and realizes the impact of it. He’s very thankful to be on it.”* |  |
| CG | | | | *“Once we felt like we had good information, of course we did some research on our own as well, then I put it to my daughter. “Would you like to proceed with the surgery, or would you prefer to give selumetinib a try?” Knowing that she could always go back and ask for the surgery at any time later. She said, “If I don’t need a surgery, I don’t want a surgery.” We decided to proceed then with the selumetinib instead.”* |  |
| **Driver of selumetinib initiation** | | | |  |  |
| PT | | | | *“I didn’t really know much about it. I just knew that it had a chance to help shrink my tumors. I don’t remember asking them any questions about it.”*  *“Just later when my parents told me about how I may have had a tracheotomy if it hadn’t been treated well, that helped me realize just how blessed I am to have gotten the medical treatment.”* |  |
| PT | | | | *“Honestly, no. I think they [parents] were onboard to begin with, and they just were trying to get it for me. They were really mad at a lot of doctors and yelling at them. (Laughter.) Until a doctor got one, they helped them.”* |  |
| PT | | | | *“I just realized my mom hugged the doctor and then we got home and then I started taking medicine. I didn’t really know what was going on. My mom hugged the doctor, I got home, and I started taking medicine. That’s all I remember happened… If I take it, I can get my dad’s phone.”* |  |
| PT | | | *“[I was] worried about trying it… [but] did not research [selumetinib].”* | | |
| **Selumetinib treatment goals** | | | |  |  |
| PT | | | | *“That I can just keep shrinking the tumor.”* |  |
| PT | | | | *“Wait. I have no idea what my goals are. Probably be in the varsity band.”* |  |
| CG | | | | *“We wanted no more tumors, but they said that this would be the best option for him to get as close as possible to what his goals were, and which was initially not to be in pain anymore and not to have these tumors everywhere that were growing bigger and bigger.”* |  |
| CG | | | | *“My goal was that the tumor would not kill him. That they would find a way for this thing to stop growing. If they could make it go away, that would be great, but at this point, we just needed it to stop growing.”* |  |
| CG | | | | *“Our goal, it’s always been quality of life, but even more so now like, “We can only shrink the tumor so much in this part, but it’s still that long-term, quality of life.” He gets to go, and he can do what he wants to do. He can participate in more sports because he feels good.”* |  |
| CG | | | | *“Our goal really was if it can control the tumor growth, that would be great. We had read and we had talked to parents whose children were in the clinical trial who had a reduction in the volume of their tumor, and so we were really hoping for something like that. He didn’t advise us to expect that. It was really just, “Let’s see if we can maintain and slow the growth at that point.””* |  |
| CG | | | | *“Just to mitigate symptoms. Obviously, we understand it’s not a cure-all, but it was just to mitigate symptoms.”* |  |
| CG | | | | *“I did not know that shrinkage wasn’t expected, I just assumed shrinkage was expected. My understanding of it through my time with CTF was that it would reduce the tumors, so she made it… I don’t want to say sound less important if it shrunk the tumors, but that the priority was to stop it from growing.”* |  |
| CG | | | | *“I think it was really about trying to alleviate pain, and stop the growth of the tumor. Just where it is it scary in the sense of it causing permanent damage, so we were thinking if we could at least halt any further growth that that would be a step in the right direction.”* |  |
| **Sources of information** | | | |  |  |
| CG | | | | *“Obviously, as his tumor started growing, I started researching. I’m on the NF Moms Rock Facebook page. People started talking about it there, and I was reading about it. I went to our neuro-oncologist with all the paperwork I had done the research about, and we decided that we would stick with the – I can’t remember the name of it now, but the other medication that he was on. I kept my eye on this one, the selumetinib trial.”* |  |
| CG | | | | *“It was presented by the oncologist, and at that time, they had the trial going on. She just mentioned it to us and basically this medication is showing good signs of actually shrinking tumors in a lot of children. We just decided to go for it and try it.”*  *“Yes, I’m actually on a Facebook group where a lot of people, their kids were participating in either a study or whatever. I was following a couple of different kids and I was seeing the difference and we decided to try it. It got minimal side effects. We were just going for it. Yes, we were following a couple of books on Facebook and yes, I would say I definitely did some research online and stuff.”* |  |
| CG | | | | *“No, actually. It was recommended by the neurologist when he was diagnosed.”* |  |
| CG | | | | *“I think I first heard about it because it was and continues to be a big deal in the NF community, being one of the only medications FDA approved for treatment.”* |  |
| **Reasons for starting selumetinib** | | | |  |  |
| CG | | | | *“I think this was the only thing they could offer us and if this didn’t work, what was next? Death? A tracheotomy or a stroke? This was a Hail Mary pass. There was no treatment, there was nothing. The surgeon said they wouldn’t touch him unless they had to.”* |  |
| CG | | | | *“He remembered that he has this, and he said, “Did you know there’s a new pill that was just approved and it could get rid of, treat, or shrink his plexiform?” I said, “No.” He gave me the name of it and said you need to find out about this. I called his neurologist the very next day and they said, “We aren’t the ones to prescribe this, but I can get you in contact with someone that can.””* |  |
| CG | | | | *“That [pain reduction] was an important component. She shared that, and I don’t remember the exact statistics, but a percentage of them were experiencing some pain relief within a relatively short period of time, and then a good number of them were experiencing tumor shrinkage as well. That was an area that we drilled in then, and we said, “Okay, so if there are areas in the body that have other tumors growing that we don’t know about, this could potentially be shrinking those as well?” She’s like, “Yes, certainly.””* |  |
| CG | | | | *“No, there aren't really any other options. There are a few different MEK inhibitors, but this was the only one that was approved, that wasn't on a trial. This was the only one that was so far approved. There's nothing else to try, actually.”* |  |
| **QoL – impact on PN growth** | | | |  |  |
| PT | | | | *“On the treatment, I remember… Because it’s a gradual change I don’t really remember when I first noticed, but I remember my family saying that it looked somewhat smaller or something when we went to a family gathering.”* |  |
| PT | | | | *“My tumors [changed]. It’s gotten smaller... I noticed in the mirror. I was happy because it got smaller.”* |  |
| CG | | | | *“One thing I noticed since she’s been on it, is it’ll be one birthmark, when she would have four or five in one area. Now it’ll just be like one or two. It’s more spread out.”* |  |
| CG | | | | *“We’re just grateful for stability at this point. The fact that it’s not growing any further, we’re happy about that. From the time frame that she stopped the trametinib to the selumetinib, there was a couple of months span there and when she went in to do another MRI, the doctor had indicated that it had grown during that 2-month period. Not very much but she could tell by the scan that it definitely had grown a little bit.”* |  |
| CG | | | | *“In fifth grade, he started, and he came home one day, and he had an afternoon snack. He goes, “You know what? Nobody asked me what was wrong with my neck this year,” and he just walked off. Like, “Nobody asks me anymore.” If I could show you a picture, which I know I can’t, you would be like, “That’s incredible.” The difference in 1 year.”* |  |
| CG | | | | *“Now that [my child] is taking [selumetinib], you can’t even see [the PN] anymore.”* |  |
| CG | | | | *“[The biggest benefit has been] the massive reduction in that PN that was visible. It was a reduction in size that was visible to the naked eye and dramatic on MRIs… I think that ultimately, it’s measurable in centimeters, not millimeters.”* |  |
| CG | | | | *“It’s been up and down. From there, it’s grown and shrunk each time we visited. Sometimes it grows, and sometimes it shrinks.”* |  |
| CG | | | | *“When he started treatment, his tumor immediately shrunk. Actually, within the first month, my husband and I and the oncologist could visibly see that it was smaller, very exciting. For the first MRI, it shrunk 20 percent and was stable for the next two MRIs.”* |  |
| CG | | | | *"Honestly, a little disappointed because I think we were really, really hopeful that it was going to start shrinking, and that we would see more of the daily benefit of [it]. I think when I feel disappointed, I try to think about if they were growing and there was nothing we could do about it, that would feel worse.”* |  |
| CG | | *“Monitoring her tumor with the MRIs, it was basically showing that it’s stable, it’s not growing. That’s a win for us. As long as it’s not growing any more, then that’s a success for us.”* | | | |
| **QoL – impact on pain** | | | |  |  |
| CG | | | | *“It just seems to me like she would itch the flat ones, but the larger ones she would be more careful with. I just assumed they hurt her.”* |  |
| CG | | | | *“I remember getting up from the table (teary) and crying to my husband in our room because I couldn’t believe he sat at the table and was able to tell us about his school day without having to complain to us that he was in pain.”* |  |
| CG | | | | *“As that thing on his lower back grew, he would have sharp, jarring, disturbing nerve pain. He would forget what he was doing in school. That physical pain, and that was probably [occurring] at age 11 through [when my child started] treatment with [selumetinib].”* |  |
| CG | | | | *“Gosh, within the first 6 weeks I would say that we had pain diminishing.”* |  |
| CG | | | | *“I realized, there was a moment, and I actually called the doctor to give him some feedback about that. I was in the yard. We were playing soccer and I kicked it, it bounced off my car and hit him in the back. He fell over because he’s so small. He fell over and then he got up, picked the ball up, and kicked it back to me. I was like, “That’s interesting.” I was like, “He didn’t take his time to get up.” I would notice things like that. He’d be playing with his cousins, and someone would shove him, and it would take him a while to get back to his feet, but he got up so quickly.”* |  |
| CG | | | | *“[The patient said], “Hey, I’m not having as much pain.” It happened… within the first 6 months where she was saying, probably about 4 months in, “I am not having as much pain in that area. I am not needing to seek out heat.””* |  |
| **QoL – impact on energy** | | | |  |  |
| CG | | | | *“He would grab a pillow and a blanket as soon as he got home from school and lay on the sofa and now…he’s more active. He doesn’t just want to be laying around. He wants to be jumping outside. He wants to be running.”* |  |
| CG | | | | *“He has what we call an ‘energy bank’. He knew how much energy he had to get through the day. He can budget his energy. What we noticed within the first few weeks of it is he would come home. He would be like, “I feel good.” I would probably even say within the first 2 weeks.”* |  |
| CG | | | | *“I called the doctor, and I was like, “He’s a lot more energetic than before, and the main thing, he doesn’t seem to be in as much pain as before.””* |  |
| CG | | | | *“Yes, his activity level has increased. He’s much more active. The pain level has been better. Running. He likes to run around. He likes to be around kids a lot more. Other things he likes to do. He’s much happier.”* |  |
| **QoL – impact on other physical aspects** | | | |  |  |
| PT | | | | *“Really good. Really good. I can sit there for hours playing my game without having to get up because I’m really uncomfortable.”* |  |
| PT | | | | *“Because I don’t know a whole lot about it. It just made it a lot harder. It was harder to walk around with a leg that had a big tumor.”* |  |
| CG | | | | *“Yes. Definitely. The pain has gotten less to a certain extent. At least he can get up to four hours of sleep a night. Let me tell you, it was bad. It was difficult. Maybe one hour and a lot of complaints and crying. Yes. Concerning where specifically on his body, I think in his back it is reduced a little, the armpits and the groin.”* |  |
| CG | | | | *“I do have a great picture of him from Christmas to August, and I have them in a split screen image. It’s amazing to me because I didn’t realize how unhealthy he looked until after the fact, but not even with the weight loss, but also, he had pretty dark circles around his eyes and hollowed out cheeks, and throat, and down into his collarbone. It’s amazing to me. When I noticed it, I happened to… We have a digital photo frame in our kitchen, and it randomly picks pictures of our Facebook and puts them up together, and it did it itself, and I was like, “Oh my God, that looks like a little refugee boy compared to my child.””* |  |
| CG | | | | *“Yes. The heart rate was initially something that we saw within the first few months. He had a really, really high heart rate but now his heart rate has gone down. It goes on the lower [side]. That’s a plus… [Also] the diarrhea [has] gone away. It’s not as prevalent as it used to be.”* |  |
| CG | | | | *“She felt like she was sleeping a little better, which makes overall mood better.”* |  |
| **QoL – impact on mental and emotional life** | | | |  |  |
| PT | | | | *“Just when I’m able to spend time with friends. Not really have to worry about… When I don’t have to stress out about school and stuff.”* |  |
| PT | | | | *“I feel like I can move normally without thinking about I have a tumor or take medicine. I feel like a normal person without taking medicine every day.”* |  |
| PT | | | | *“I think something good about it is that I get to live without the stress of knowing that I might die of suffocation.”*  *“There's not a really easy way for someone to feel like they're experiencing what you are when you've gone through as much as I have. Because it gets really tough sometimes, so it's hard for someone to go through all the little parts that come with it, but it's also all the big parts… What's hard about it is worrying about what's going to align in the future. Not knowing.”* |  |
| CG | | | | *“He’s not as moody. He’s a regular moody teenager but before I think it was more because he was in so much pain, it would just come out when he was talking to all of us. Now he’s just like normal, moody, teenage stuff.”* |  |
| CG | | | | *“He’s definitely happier than before. He can’t even feel the changes in his body. He’s more upbeat. It’s not like he’s out there wrestling and doing all that, playing with dirt bikes or anything, but he’s definitely a bit happier than before.”* |  |
| CG | | | | *“Now that we are medically stable, let’s go back and look at this stuff here… I said, “He’s not having anxiety, he’s not upset…” Because of that, we took him off of [an anxiety medication]. He’s been fine.”* |  |
| CG | | | | *“He can be out in public more. He feels confident because the medication is helping him. He does feel confident, yes. His stomach is not bothering him so much like it used to. He’s had a little stomach pain, but he’s able to go out in public and know that he’s being taken care of. The medication is making him feel like he’s making progress.”* |  |
| CG | | | | *“I think it’s made him have a more positive outlook on life. He’s more confident in being active and associating with others. It makes him less insecure about his appearance and his ability to function. The support is also important as well. All of those have helped him manage his situation.”* |  |
| CG | | | | *“I think her quality of life has definitely improved… We actually got her to go to a class that had me and her dad and a couple other families... I think it’s definitely improved enough that she feels like she doesn’t want to be at home and so clingy, as much.”* |  |
| CG | | | | *“I think, though, not being in pain leaves her more confident, more engaged. She’ll participate in a few more activities, whereas before she’d be like, “I’m hurting. I’m going to sit this one out.” When she was fighting the pain without an answer for a plan of what we were going to do, she probably tended to see things in a more negative light, not feel hopeful, be very – I don’t want to say hypochondriac, but everything fed into the problem she was experiencing.”* |  |
| **QoL – impact on social and learning domains** | | | |  |  |
| PT | | | | *“I can sit a lot more longer. [It helps me with] school.”* |  |
| PT | | | | *“It does [help me with school]. At my old school, I used to have a ball because my butt was really bothering me back then. Now, it’s definitely [better]. I can sit there for hours without having it.”* |  |
| PT | | | | *“I go to school. I don’t have so much pain anymore. It’s easier to study, and also a lot easier to hang out with my friends.”*  *“Whenever I play games with my friends, or talk to them, it’s just easier to talk to them, hang out with them. We’ll work on stuff without the pain and getting tired from the pain.”* |  |
| CG | | | | *“He wants to be playing with his brothers. He wants to be doing stuff that normally he hadn’t been doing for quite some time.”* |  |
| CG | | | | *“Some of [the improvement] is selumetinib… What we thought is, “Okay, it takes the pain away. He’s able to concentrate more in school…” The pieces are coming together. The connections are being made.”* |  |
| CG | | | | *“It has reduced the frequency and, I guess intensity might be the right word...* *It's all about self-esteem with these girls, especially when they're in high school. It has helped me save a lot of money on foundation, so she doesn't have to cover [the tumor] up.”* |  |
| CG | | | | *“We’ve also spoken with some people who have told us that there is some research going on and I believe there is possibly a connection with NF students who do have struggles with reading and academic concerns, also showing a benefit after starting selumetinib in the areas of academic concern. If that proves to be true, we know we certainly saw substantial gains for him after starting. If that proves to be, then I would say that was definitely another benefit for him.”* |  |
| CG | | | | *“He’s making an effort to actually mingle with them [cousins]. He actually makes an effort, yes. He tries. He’s still not exactly up to speed, but… His cousins still know.”* |  |
| CG | | | | *“His speech pathologist has been pretty happy with his progress. He’s improving a lot. Overall, teachers have also been pretty positive about his improvement as well the last few years. He’s moving along great. That bodes well.”* |  |
| CG | | | | *“I’ve seen improvements and struggle times throughout. As you move through the years it gets harder, but I can’t directly tie selumetinib to a change, but I would say that the reduced anxiety would contribute to more successes in school, for sure, but it would be hard to measure at this point.”* |  |
| **Most important benefit of taking selumetinib** | | | |  |  |
| PT | *“…just the size and the pain are, I would say, the main ones”* | | | | |
| PT | *“It’s got a little easier, but it’s stayed the same a bit.”* | | | | |
| CG | | | | *“[The most important benefit is] that my son, he feels confident. He knows there’s something out there that can help him because no child wants to be left alone when you’re diagnosed with something, you’re sick, and there’s nothing that I can do to help you. You can have your mother and father, but there’s no medication. I can’t take you to the doctor to help you. I think knowing that there is a medication that can help him is very beneficial. Yes, it makes him feel normal again.”* |  |
| CG | | | | *“Arresting the growth of the tumor and getting it shrunk, and I don’t know when we’re going to look into this, because I don’t know if it has anything to do with his age, but it may be possible to get that PN shrunk to the point where plastics would be able to go in and do surgery, which has never been an option in the past. If it could have us on a pathway to have a partial resection or debulking, that would be pretty awesome.”*  *“I have all positive things to say. This was a drug that had enormous benefit. If you look at this, this is almost all upside in my estimation. It would be interesting to hear what he said. I would tell people to run, don’t walk and get it. Because it made such a difference in terms of the pain, the growth, all of that. The other, those secondary benefits on the other fibromas were just a bonus.”* |  |
| CG | | | | *“I think when he tells me that he’s not in pain [is the biggest benefit]. When he told me that, I think that made our decision to pick going this route the best one we could because we didn’t even know that that was such an issue. We didn’t know exactly how much pain he was in because he couldn’t verbally tell us that it was pain because he just thought it was normal.”* |  |
| CG | | | | *“I think the most important benefit is that because we were able to start taking [selumetinib] before the clinical impact, it prevented him from having a tracheotomy. They know he would have probably had a tracheotomy within a year or two, the way his airway was starting close over. That was their main concern, but then his carotid arteries are also encapsulated. It was so slow growing that they’re not constricted, but if it had kept growing, who knows? In one of his radiology reports, it says that his mandible was starting to erode. The bottom of his jaw, they said, was starting to impact his jawbone. If it had kept growing, it could have affected his jaw. It could affect his motor movement, his swallowing, his breathing, his carotid artery… There’s a lot of stuff in your neck (laughs). It was just taking up all that space in his neck. He had some movement, mobility issues where he couldn’t turn his neck a lot. I will mention that. He has full movement of his neck now. That’s not a problem anymore.”* |  |
| CG | | | | *“Phew. The heart issues, I would say is the most important thing. You don’t want your child to die early from heart complications. That’s been able to be mitigated a lot is the most important thing, but from the kid’s perspective, they care more about the physical appearance as far as… When you interact with other kids, it’s important to them. That’s very important to them, that visual appeal. I think that’s also important as well.”* |  |
| CG | | | | *“The pain relief, of course. I think knowing that it’s targeting not just the one – versus surgery, the selumetinib is working throughout her whole body for things we don’t even know about, which might give her some long-term benefit that we can’t even measure because we don’t know what would develop. The immediate benefit is the reduction in pain. The long-term benefit is that it’s helping throughout her body… I think that long-term benefit of trying to give her whole body the best opportunities we can is reassuring.”* |  |
| CG | | | | *“[The most important benefit is] that we’re not seeing growth. It’s so hard to say because we don’t know what would happen if we weren’t on it, but if we can keep this one under control, that’s the benefit that we’re hoping for.”* |  |
| **Treatment goals since initiating selumetinib** | | | |  |  |
| CG | | | | *“He’s alive. That’s what I always say. He’s alive. He’s 17 and he’s thriving. He’s a 4.1 GPA honor student and he’s with us and thriving and he’s a great value to our family and everybody around him. He’s a miracle and the drug is our miracle. He wouldn’t be here. If he were here, his quality of life would not be good at all. He would be in a very chronic situation of either a vent or a tracheotomy or other things that would have come to pass by now if he were even alive then.”* |  |
| CG | | | | *“I don’t know. We were there last Wednesday, and I asked her, I said, “You know, he’s 16. This is only ‘recommended’ for 18-year-olds and under. What happens in two years?” She said, “We’re not going to discuss that now because we don’t know.” She said, “If he needs to be on this still, I’m sure he can be on this still,” and she just gave me it’ll-be-fine look. I don’t know. It’ll just be one of those wait-and-see types of things.”* |  |
| CG | | | | *“At this point, our NF doctor wants him on selumetinib for two years, and then they want to wean him off and then recheck to see if the tumor stays ‘Dormant,’ is how she refers to it. Because it has shrunk so much, if it stays dormant, then we would go to 6-month and yearly MRIs. If it were to start growing again, then we would continue on selumetinib.”* |  |
| CG | | | | *“I’m just hoping that the medication just stays stable because it’s actually helping him stay stable. He’s continuing to improve. As long as there is the same medication, nothing different, same dosages, which is 25 milligrams, or milliliters orally, then I don’t see any changes. I just see him slowly but surely progressing. I see this being a benefit. I’m very thankful for the medication.”* |  |
| CG | | | | *“At the beginning we had a pretty clear goal of doing this for a couple of years and see how it turns out. Of course, that in the context of knowing that NF is progressive and is going to be changing all the time, we’ve never set a goal of specifically saying, “Well, we have to see this result or else.” It’s more, “Let’s see where it goes.” If it postpones or indefinitely postpones some surgeries, then we’ve certainly met a goal. Even postponing it for the short term has been meeting that goal for sure.”* |  |

Abbreviations: CG, caregiver; CTF, Children’s Tumor Foundation; ENT, ear, nose and throat; FDA, Food and Drug Administration; IP, individual plan; MEK, mitogen-activated protein kinase kinase; MRI, magnetic resonance imaging; NF, neurofibromatosis; NF1, neurofibromatosis type 1; OT, occupational therapy; PN, plexiform neurofibroma; PT, patient; QoL, quality of life.

## Supplementary Table 5. Caregiver-reported Initial Signs/Symptoms of NF1, in Addition to PN

| **Initial symptom** | ***N* = 19 (%)** |
| --- | --- |
| Café-au-lait spots | 16 (84.2) |
| Pain | 14 (73.7) |
| Freckling | 11 (57.9) |
| Learning disabilities | 9 (47.4) |
| Fine motor skills issues | 8 (42.1) |
| Sleep issues | 8 (42.1) |
| Speech issues | 7 (36.8) |
| Lisch nodules | 7 (36.8) |
| GI issues | 5 (26.3) |
| Exhaustion | 5 (26.3) |
| Behavioral issues | 4 (21.1) |
| Walking delay | 4 (21.1) |
| Itching | 4 (21.1) |
| Vision problems | 3 (15.8) |
| Bone deformities | 2 (10.5) |
| Glioma | 2 (10.5) |
| Cardiovascular problems | 1 (5.3) |
| Hypotonia | 1 (5.3) |

Abbreviations: GI, gastrointestinal; NF1, neurofibromatosis type 1; PN, plexiform neurofibroma.

## Supplementary Table 6. Descriptions of the Selection of Images Chosen by (a) Patients and (b) Caregivers Representing Life Before Patients Started Selumetinib

**A** Patients

| **Description of picture selected by patients (picture number)** | **Number of times chosen (*N* = 10)** |
| --- | --- |
| Lightning (4) | 3 |
| Traffic (9) | 2 |
| Rollercoaster (3) | 2 |
| Brick wall (5) | 1 |
| Rainy window (11) | 1 |
| Skyscrapers (12) | 1 |
| Wave (13) | 1 |

**B** Caregivers

| **Description of picture selected by caregivers (picture number)** | **Number of times chosen (*N* = 18)** |
| --- | --- |
| Rainy window (11) | 6 |
| Lightning (4) | 3 |
| Tornado (2) | 2 |
| Rollercoaster (3) | 2 |
| Fireworks (1) | 1 |
| Burning house (6) | 1 |
| Lake and rainbow (7) | 1 |
| Red palm trees (8) | 1 |
| Traffic (9) | 1 |
| Cactus (14) | 1 |
| Blue palm trees (16) | 1 |
| Brick wall (5) | 1 |

## Supplementary Table 7. Descriptions of the Selection of Images Chosen by (a) Patients and (b) Caregivers Representing Life After Patients Started Selumetinib

**A** Patients

| **Description of picture selected by patients (picture number)** | **Number of times chosen (*N* = 9)** |
| --- | --- |
| Red palm trees (8) | 3 |
| Rollercoaster (3) | 2 |
| Blue palm trees (16) | 2 |
| Fireworks (1) | 1 |
| Lake and rainbow (7) | 1 |

**B** Caregivers

| **Description of picture selected by caregivers (picture number)** | **Number of times chosen (*N* = 18)^a^** |
| --- | --- |
| Rollercoaster (3) | 4 |
| Blue palm trees (16) | 3 |
| Traffic (9) | 3 |
| Fireworks (1) | 2 |
| Cactus (14) | 2 |
| Lightning (4) | 2 |
| Red palm trees (8) | 2 |
| Rainy window (11) | 1 |

# **Supplementary Figure**

## Supplementary Figure 1. Caregiver-reported most important benefits of taking selumetinib.


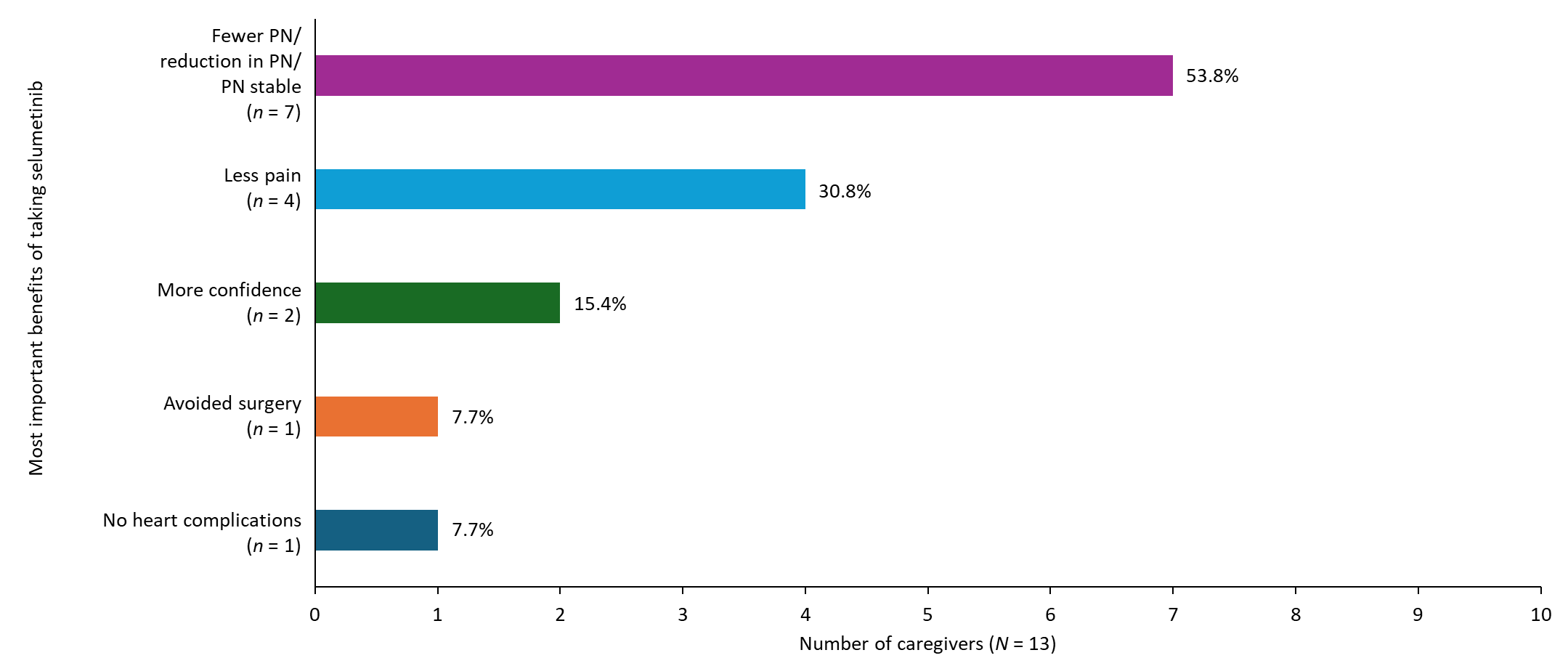


Abbreviation: PN, plexiform neurofibroma.

# **Plain Language Summary**

People with neurofibromatosis type 1 (NF1) can develop tumors called plexiform neurofibromas (PN). PN are often painful, and can have a notable impact on children with NF1 and their caregivers. Selumetinib is a drug that is approved by U.S. Food and Drug Administration for the treatment of children over the age of 2 years with NF1 and symptomatic, inoperable PN. The aim of this study was to better understand the experiences of children with NF1‑PN before and after selumetinib treatment from the perspective of the patients and their caregivers.

Children participated in 30-minute one-to-one telephone interviews depending on their age and ability to express themselves; they were asked about their NF1-PN diagnosis and symptoms, and their experiences with selumetinib. Caregivers participated in 45-minute one‑to-one telephone interviews designed to enable reflection on their child’s diagnosis and symptoms, and the decision to start selumetinib, and experiences with selumetinib.

Before starting selumetinib, PN negatively affected the lives of these children. Caregivers primarily chose to start selumetinib in their children to stabilize/shrink PN and reduce their child’s pain. Most caregivers reported PN stability/shrinkage, reduced pain, and improved mental and emotional wellbeing in their children once they had started selumetinib treatment, and their children generally agreed that selumetinib had improved their quality of life.
